# Using Baclofen to Explore GABA-B Receptor Function in Alcohol Dependence: Insights From Pharmacokinetic and Pharmacodynamic Measures

**DOI:** 10.3389/fpsyt.2018.00664

**Published:** 2018-12-14

**Authors:** Claire F. Durant, Louise M. Paterson, Sam Turton, Susan J. Wilson, James F. M. Myers, Suresh Muthukumaraswamy, Ashwin Venkataraman, Inge Mick, Susan Paterson, Tessa Jones, Limon K. Nahar, Rosa E. Cordero, David J. Nutt, Anne Lingford-Hughes

**Affiliations:** ^1^Neuropsychopharmacology Unit, Division of Brain Sciences, Department of Medicine, Centre for Psychiatry, Imperial College London, London, United Kingdom; ^2^Toxicology Unit, Imperial College London, London, United Kingdom; ^3^Centre for Brain Science, University of Auckland, Auckland, New Zealand

**Keywords:** baclofen (PubChem CID: 2284), addiction, pharmacodynamic (PD), pharmacokinetic (PK), alcohol use disorder (AUD), GABA-B receptor, EEG, growth hormone

## Abstract

**Background:** The role of GABA-B neurotransmission in addiction has recently received increased attention, with clinical trials indicating that baclofen, a GABA-B receptor agonist, may reduce alcohol consumption, craving and promote abstinence. However, the optimal dose to treat alcohol dependence is unclear with patients requesting and tolerating much higher doses of baclofen, compared with other clinical uses. We assessed the pharmacokinetics and pharmacodynamics (PK/PD) of baclofen to provide insight into GABA-B sensitivity in this patient group, relative to controls.

**Methods:** Male healthy volunteers (controls, *n* = 12) and abstinent alcohol dependent individuals (AD, *n* = 8) received single oral doses of baclofen or placebo in a 3-way crossover design. Controls received placebo/10 mg/60 mg baclofen in a randomized, double-blind design, AD received placebo/60 mg/90 mg baclofen in a single-blind design. PK/PD measures were recorded at baseline and multiple time-points up to 6 h post-dosing, including plasma baclofen, plasma growth hormone (GH), Subjective High Assessment Scale (SHAS) and biphasic alcohol effects scale (BAES). Repeated measures ANOVA analysis explored “change from baseline” dose, time, group, and interaction effects, *t*-tests compared peak effects.

**Results:** Dose-dependent effects of baclofen on PK and PD measures were observed in both control and AD groups. Whilst there were no significant group differences in any baclofen PK parameters (*t*_1/2_, *t*_max_*, C*_max_*, AUC*), marked differences in PD effects were clearly evident. In controls, 60 mg baclofen significantly increased total SHAS and BAES scores, and significantly increased plasma GH levels compared with placebo, with peak effects at 60–120 min, in line with its PK profile. In AD, 60 mg baclofen had limited effects on these parameters; SHAS scores, BAES scores and plasma GH levels were significantly blunted compared with controls (significant group^*^time interactions *P* = 0.0014, 0.0015 and *P* < 0.0001, respectively).

**Conclusions:** Our study shows blunted sensitivity to baclofen in AD relative to controls, with no difference in PK suggesting a lower GABA-B receptor sensitivity. This may explain why higher baclofen doses are requested and tolerated in the treatment of alcohol dependence. Our data has implications for choice of dose in future clinical trials in AD and possibly other substances of dependence.

## Introduction

The GABA-B receptor plays a central role in the control of neurotransmitter release and there is good evidence from preclinical studies that modulation of this receptor modifies the brain's reward process and mesolimbic dopamine release (1–3). Preclinical animal studies have shown that GABA-B agonists (e.g., baclofen) attenuate many of the positive reinforcing effects of reward, especially to drugs and alcohol (4–8).

Baclofen is the only selective GABA-B agonist available for use in man. Based on the promising preclinical evidence, its potential to treat alcohol/drug dependence has been investigated. Baclofen is currently licensed for the treatment of spasticity in neurological conditions. However, there is widespread off-label use in the treatment of alcohol dependence, particularly in relapse prevention (9–11). Open-label studies and clinical trials have shown that oral baclofen at doses of 30–80 mg/d can increase rates of abstinence (12, 13), reduce alcohol craving (13–15) and anxiety (14–16), with good tolerance and few side effects (17). Such studies have resulted in increasing off-label use of baclofen in many parts of the world for the treatment of alcohol dependence, particularly for those with higher severity and anxiety levels (9–11, 18). Its widespread use in France led to “Temporary Recommendations for Use” in alcohol dependence in 2014. Marketing Authorization Approval was recently granted by the French National Agency for the Safety of Medicines and Health Products (ANSM) for doses up to 80 mg daily. More recent studies however, have failed to demonstrate a superior clinical outcome of baclofen to maintain abstinence at similar doses (30–60 mg/d), compared with placebo. This may have been due to an increased proportion of participants with milder levels of dependence (19–21). Of note, one study showed a beneficial effect only in those with higher levels of comorbid anxiety (19).

The foremost and critical debate concerning the use of baclofen to treat alcohol dependence has centered on what is the appropriate dose. In the first clinical studies, 10 mg three times a day (30 mg/d) was chosen “based on the minimum therapeutic dosage at the fractioning modality recommended by the drug manufacturer to avoid side effects” (22). Thus the dose of baclofen used in these early clinical studies was determined by its license for, and experience in, treating of muscle spasticity in neurological conditions. However, a case report described using 270 mg/d of baclofen to successfully treat alcohol dependence and to suppress craving and anxiety. The chosen dose was based on neurologists' use of baclofen up to 300 mg/d and the translation of preclinical doses to the clinic (23). Following this report, much higher doses of baclofen began to be requested and prescribed (up to 300 mg/d).

Accordingly, more recent trials included doses of up to 270 mg/d, although the mean dose achieved was generally much lower (24–26). Of these, only one study reported that baclofen was superior to placebo in maintaining abstinence, though no dose-response effects or benefit in craving or anxiety were observed (24). The effective comprehensive psychosocial programme in another of the studies likely reduced the potential for any additional effect of baclofen (25). In the French trial, it was speculated that public debate about taking baclofen whilst drinking impacted on abstinence rates though a “tendency toward a reduction in alcohol consumption and a significantly decreased craving” (26). Whilst trials of higher doses of baclofen generally reported good tolerability and safety, concerns have been expressed about adverse effects at such high doses, particularly when taken with alcohol (27–29).

In the absence of a robust biomarker to assess the efficacy and optimal dose of baclofen to treat alcohol dependence, it is therefore important to understand if the pharmacokinetics of baclofen and/or the sensitivity of the GABA-B system itself is altered in alcohol dependence. In healthy individuals, a linear plasma concentration-dose relationship has been reported after 10–40 mg of baclofen (30–33). However baclofen plasma levels have been reported to be highly variable in alcohol dependent individuals at a range of doses [30–240 mg; (34)]. Concerning the pharmacodynamic effects of baclofen, changes in sedation, impulsivity, EEG markers and psychomotor performance have been assessed in healthy controls at low, i.e., 10–30 mg, doses (32, 35–39). Studies in abstinent alcohol dependent individuals reported blunted baclofen induced growth hormone (GH) release compared with healthy controls (40–44). This blunted release was evident at low doses of baclofen (10–20 mg) but there are no similar studies using higher doses, nor were plasma levels measured.

Despite interest in the clinical use of baclofen in alcohol dependence, and debate around optimal dose, there is a paucity of human laboratory studies characterizing its combined pharmacokinetic (PK) and pharmacodynamic (PD) effects. We therefore explored these measures further by comparing the effects of a single dose of baclofen (10, 60, or 90 mg) with placebo on a range of objective and subjective measures, whilst measuring plasma levels in healthy volunteers (controls) and abstinent alcohol dependent (AD) individuals.

## Methods

This was a randomized, placebo controlled, three-way crossover study, in which control and abstinent AD participants received a single oral dose of 10, 60, or 90 mg baclofen or placebo (ascorbic acid 100 mg). Study days were separated by at least 1 week and were conducted at the National Institute of Health Research Clinical Research Facility (NIHR CRF) at Imperial College Healthcare NHS Trust, Hammersmith Hospital. Studies were approved by UK National Ethics (NRES) Committees (London-Chelsea, REC number; 11/LO/1973 and West London & GTAC, REC number; 15/LO/1000, for control and AD studies, respectively), and were carried out in accordance with Good Clinical Practice Guidelines and the principles outlined in the Declaration of Helsinki.

### Participants

Healthy male participants (controls, *n* = 12), and male abstinent alcohol dependent (AD, *n* = 8) participants were recruited via advertising, volunteer databases, or through local NHS addiction services and other partner organizations. Dependent individuals met DSM-5 criteria (45) for severe alcohol use disorder, were abstinent for at least 4 weeks prior to study sessions and had never met dependence criteria for other substances (excluding nicotine). Healthy controls were recruited who had no history of drug or alcohol dependence (except nicotine). Exclusion criteria included current use of psychoactive medication (including benzodiazepines, antidepressants and relapse prevention medication), current primary axis I diagnosis, past history of psychosis, past history of enduring severe mental illness. The higher doses of baclofen (60 and 90 mg) used in this study were predicted to produce substantial subjective effects, some of which are similar to those of alcohol. For this reason, we deliberately recruited healthy subjects who were regularly drinking between 8 and 160 g (1–20 UK units, i.e., within recommended safe limits from UK Chief Medical Offer guidelines at the time of testing) of alcohol per week (Table 1), and thus were familiar with such central effects. Two of these control participants scored 8–9 on the Alcohol Use Disorders Identification Test (AUDIT) and one participant scored 20 (though stated he had recently reduced his alcohol intake). Careful screening by a psychiatrist with addiction expertise, found no evidence of current or previous alcohol use disorder in these participants. There were two separate cohorts of controls recruited. Cohort one was recruited first (*n* = 9) in order to establish the PK-PD protocol, and consisted of young male volunteers (average age 24.7 years). Cohort two (*n* = 3, average age 52.0 years) was recruited alongside the AD cohort (average age 53.1 years), in order to provide a better age match, and to allow higher doses of baclofen to be explored. There were no PK or PD differences in response to baclofen between young and old controls, and no correlation between age and PD variables, so control data were collapsed across cohorts, unless otherwise stated.

**Table 1:** Demographic variables.

	**Healthy control, *n* = 12**	**Alcohol dependent, *n* = 8**
Age (range)	31.5 ± 13.2 (21-56)	53.1 ± 8.9^*^ (39-63)
BMI	23.8 ± 5.1	31.3 ± 4.9^*^
Current smokers (n)	0	4
Cig/day	–	11.3 ± 14.4
Fagerstrom	–	7.0 ± 2.3
AUDIT (range)	7.8 ± 5.6 (2-20)	–
Alcohol intake (g/week)	67.2 ± 42.4	–
Months abstinent (range)	–	34.9 ±27.0 (8-72)
Lifetime ‘high risk' alcohol exposure (years)	–	21.1 ± 11.8
Beck Depression Inventory	3.2 ± 4.7	4.1 ± 3.9
STAI (trait anxiety)	32.2 ± 9.2	36.1 ± 8.6
Locus of Control Scale	6.3 ± 2.0	6.4 ± 2.9

*Values are mean ± S.D. unless otherwise stated*.

* *P < 0.05 unpaired t-test*.

Following informed consent, subjects attended a screening visit which included taking medical, psychiatric and alcohol use history, a physical examination, routine blood hematology/biochemistry and electrocardiogram. Participants also completed the Fagerstrom test for nicotine dependence (46), Spielberger Trait Anxiety Inventory (STAI), Beck Depression Inventory (BDI (47) and Locus of Control Scale (48). A time-line follow-back was completed to calculate lifetime alcohol exposure in the AD cohort. High-risk alcohol exposure was calculated as lifetime cumulative weeks with greater than 60 g average daily alcohol consumption, converted into years [according to WHO guidelines, (49)].

### Procedures

On each study day, eligibility was checked, including a negative urine drug screen and alcohol breath test. Caffeine intake was permitted but was matched across study days. All participants had breakfast and were provided with a similar lunch. An intravenous cannula was inserted for blood sampling (for baclofen and GH levels) and electrodes were applied for EEG monitoring. Objective and subjective measurements were variously undertaken at baseline (prior to baclofen dosing) and at 30, 60, 120, 180, 240, and 360 min after dosing, including measures of vital signs, rating scales, psychomotor performance and adverse events. Due to the time to complete certain measures (e.g., EEG), the subjective questionnaires were performed 15–20 min later than the times indicated. Participants were allowed to smoke *ad-libitum*, to reflect their typical cigarette use, so were not in overt withdrawal during behavioral or EEG measurements. Smoking breaks were taken at the same time across sessions, wherever possible.

Following completion of baseline measures, controls were dosed with either oral placebo, 10 or 60 mg baclofen in a randomized, double-blind cross-over manner. In the first control cohort, 9 participants received placebo, 8 received 10 mg baclofen and 9 received 60 mg baclofen. In the second control cohort, 3 received placebo, 3 received 60 mg and 1 received 90 mg baclofen. The 90 mg baclofen dose was administered in a single-blind manner, but the effects were not well tolerated, so this test dose was abandoned in further control participants.

The AD group were dosed with placebo, 60 or 90 mg baclofen in a randomized, single-blind cross-over manner. In this case a pseudo-randomization was adopted in order to ensure that participants could tolerate the 60 mg dose of baclofen, prior to receiving the higher 90 mg dose. Researchers were aware of the pseudo-randomization but participants were not, so remained fully blinded. In hindsight, because of the greatly increased tolerance to baclofen in this group, the need for pseudo-randomization was unwarranted. In the AD cohort, 8 received placebo and 60 mg baclofen, and 6 received 90 mg.

### Drugs

Drugs were supplied by Hammersmith Hospital Pharmacy and comprised of baclofen (10 mg, white scored tablets, or ascorbic acid (vitamin C, 100 mg, white scored tablets). Drugs were stored within the NIHR CRF and prescribed by the study doctor according to the randomization on the day of testing. To maintain blinding, drugs were administered by a study nurse who had no further part in the study. All participants were blindfolded and took their tablets with plenty of water. In controls, 6 tablets were administered (6x vitamin C, 6x baclofen 10 mg, or 1x baclofen 10 mg with 5x vitamin C. The AD group took 9 tablets (9x vitamin C, 9x 10 mg baclofen or 3x vitamin C with 6x 10 mg baclofen).

### Blood Sampling and Assays

Blood samples were taken from the venous cannula at *t* = 0 (prior to baclofen dose), then at +30, 60, 120, 180, 240, and 360 min time points post baclofen dose and stored on ice. Plasma was separated by cold centrifugation and stored at −30°C for later analysis.

#### Growth Hormone Assay

Plasma GH levels were measured (μg/L) using a standard IMMULITE®2000XPi solid-phase, two-site chemiluminescent immunometric assay system with a sensitivity of 0.05 μg/L. Analyses were carried out by the Clinical Biochemistry Department, Imperial College Healthcare NHS Trust, Charing Cross Hospital, London, UK.

#### Baclofen Assay

Plasma concentrations of baclofen were measured using the liquid chromatograph mass-spectrometry (LC-MS) method as previously described (50), at the Toxicology Unit, Imperial College London.

### Pharmacokinetics

Pharmacokinetic parameters were calculated by modeling the data as follows: plasma concentrations (C_P_) of baclofen at time *t* were fitted using a non-linear least squares algorithm to a first-order absorption-elimination model (absorption constant; k_a_, elimination constant; k_e_, mass of drug administered x fraction of drug absorbed; *X*_a_) to calculate the following parameters: peak plasma concentration (*C*_max_) and time to reach *C*_max_ (*t*_max_) (both solved graphically), half-life (*t*_1/2_) (ln 2/k_e_ and area under the curve (*AUC*) for the duration of the recording, according to the formula:

(1) $$\begin{array}{r} C_{P}\left(t\right)=\frac{X_{a}\cdot k_{a}}{k_{a}-k_{e}}\left(e^{-k_{e}\left(t\right)}-e^{-k_{a}\left(t\right)}\right) \end{array}$$

Further, the relationship between plasma levels of baclofen and the EEG response were estimated using a simple two-compartment (or one-tissue-compartment) model, which is incomplete but practical and suited to the data available. If the EEG response is assumed to be directly coupled to the mass of baclofen in the brain, then the brain is treated as a single compartment and only uptake (*K*_1_) and washout (*k*_2_) rates are needed to describe the change in EEG power (*P*_*EEG*_) (see Equation 2).

(2) $$\begin{array}{r} \frac{dP_{EEG}\left(t\right)}{dt}=K_{1}C_{P}\left(t\right)-k_{2}P_{EEG}\left(t\right) \end{array}$$

Solving this equation, we fit *P*_*EEG*_ over time using a convolution integral of the plasma model with a monoexponential curve (Equation 3), using a non-linear least squares optimizer.

(3) $$\begin{array}{r} P_{EEG}\left(t\right)=C_{P}\left(t\right)⊗K_{1}e^{-k_{2}t} \end{array}$$

### Objective Measures

Heart rate and blood pressure observations were made using a Phillips SureSigns VM4 monitor. The “Zig-Zag” pencil and paper maze test of motor coordination and sedation was administered at all time-points except 30 min post dosing [Zig-Zag Tracking task; (51)].

EEG was acquired using the Neuroscan (Compumedics) system, with a sampling rate of 1,000 Hz. A total of 24 electrodes were placed according to standard 10–20 criteria, and EEG was recorded for 5 min with eyes open and 5 with eyes shut at each time point, using Cz as the common reference. During recording, subjects were seated upright in a reclining chair and were asked to try and stay awake. If they fell asleep during eyes closed they were allowed to doze but were woken if the EEG showed sleep during the eyes open period. In the control group, for the placebo and 60 mg conditions *n* = 7 recordings were available for analysis (3 data sets were lost due to interference & technical issues). For the 10 mg condition a further data set was lost (due to drop-out), resulting in *n* = 6 overall. In the AD group, *n* = 6 recordings were collected, however, these were not further analyzed after visual inspection (see below results).

Offline analysis was performed visually and automatically for each recording. We first carried out visual scoring of all recordings using a scale for daytime sleepiness previously used by our group (52), which uses the presence of eye movements, muscle activity, alpha and theta activity to assign a score to each 15 s' activity. Data were examined visually for gross artifacts (e.g., head movements) and sections containing these were removed. Because data from periods with eyes open included copious eye movement and muscle artifacts, we excluded the electrodes Fp1, Fp2, F8, F7, A1, A2 from the analysis, leaving 15 recording electrodes. The data were then globally re-referenced (excluding electrodes outlined above, Cz and any channels identified as poor) and segmented into epochs of 2 s in length. Using the FieldTrip toolbox, a low pass filter was applied (35 Hz) and any bad channels interpolated using the spline method. Frequency analysis was conducted using Hanning windowed fast Fourier transforms between 1 and 50 Hz at 0.5 Hz frequency steps. For statistical analysis, theta (4–8 Hz) band activity was extracted from individual spectra (53, 54). We did not investigate the delta spectra as we were unable to accurately remove all eye movements which contaminated this band. Pre-dose baseline recordings were subtracted from each time-point to give change from baseline, and then paired contrasts were performed between placebo and 60 mg baclofen using permutation testing of t statistics (54). False discovery rate (FDR) across multiple channels with each comparison was controlled using resampling, with 50,000 permutations for each map.

### Subjective Measures

Participants completed the following rating scales at all time points: Subjective High Assessment Questionnaire [SHAS, (55)], Drinking Expectancy Questionnaire [DEQ, (56)], Biphasic Alcohol Effects Scale [BAES; (57)], and verbal visual analog scales (VAS) using a visual prompt (0–100) for Sleepy, Relaxed, Tense and Alert.

### Data Analysis

All statistical analyses were carried out using Graphpad Prism, version 7.00 or IBM SPSS (version 24). For graphical representation and statistical analyses, raw data were converted to change from baseline (CB) with the exception of plasma baclofen levels. In a small number of missing value cases, data were interpolated using the average of time points either side of the missing data. Repeated measures ANOVAs were used to explore effects of dose, time and interaction for within-subjects comparisons in those participants who were able to complete *all* measures for that variable, with Tukey's multiple comparison *post-hoc* test to determine significant dose effects at each time-point. Mixed ANOVAs were used to explore effects of group, time and interaction across all subjects, as appropriate, with Sidak's multiple comparison *post-hoc* test to determine significant group effects at each time-point. Paired and unpaired *t*-tests were conducted to determine dose and group differences, respectively, at peak baclofen concentrations (*t* = 120 min time-point). Correlations were based on Spearman's rho or Pearson correlation coefficients. For GH data, all non-determinable values (i.e., those that fell below the limit of detection of 0.05 μg/l) were replaced with 0.05/2 (i.e., 0.025) to avoid data skew.

## Results

### Demographics

Control participants were significantly younger than AD patients, with lower body mass index, and there were fewer current smokers in this group (Table 1). There were no significant group differences between depression, anxiety and locus of control scores.

### Pharmacokinetics

**Figure 1:**
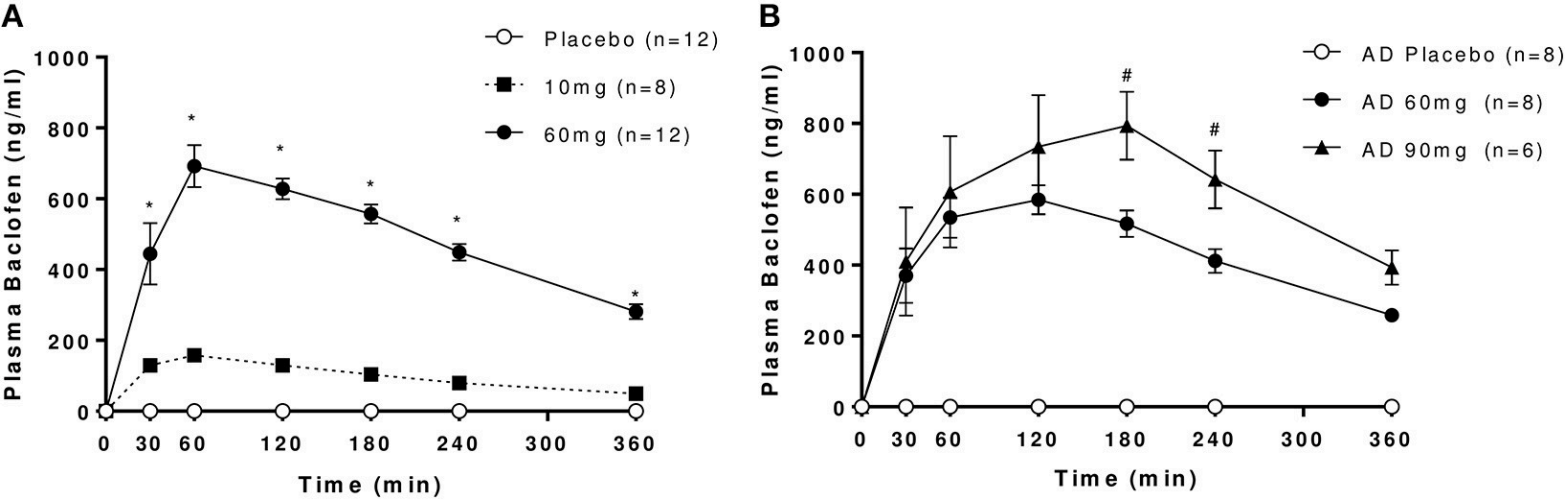
Plasma levels of baclofen following oral administration of 10, 60, or 90 mg baclofen or placebo in **(A)** controls and **(B)** AD. Data are mean ± S.E.M. Repeated measures ANOVA revealed significant effects of time, dose and dose by time interaction in controls (*n* = 8): Time: *F*_(6, 42)_ = 58.1, *P* < 0.0001, Dose: *F*_(2, 14)_ = 227.7, *P* < 0.0001, Dose × Time: *F*_(12, 84)_ = 43, (*P* < 0.0001) and in AD (*n* = 6): Time: *F*_(6, 30)_ = 9.5, *P* < 0.0001, Dose: *F*_(2, 10)_ = 122.6, *P* < 0.0001, Dose × Time: *F*_(12, 60)_ = 5.7, (*P* < 0.0001). ^*^60 mg > 10 mg, ^#^90 mg > 60 mg (*P* < 0.05, Tukey's multiple comparison test).

Plasma levels increased in a dose-dependent manner following oral administration with baclofen in both controls and AD (Figure 1). No baclofen was detected in plasma samples after placebo. Good separation was observed between 10 and 60 mg baclofen doses in controls, and between 60 and 90 mg doses in AD, although there was considerably more variability at the higher doses in both groups. Significant increases in *C*_max_ values were observed with increasing dose (Table 2). Modeled data revealed that there was a significant increase in *C*_max_ with increasing dose in both groups as expected. An increase in *t*_max_ was evident between 10 and 60 mg baclofen in controls, where *t*_max_ occurred approximately 30 min later (62 and 88 min for 10 and 60 mg, respectively). *t*_max_ was similarly increased following 90 mg compared with 60 mg in AD (135 and 102 min, respectively). There were no significant differences in any PK parameters between controls and AD following the 60 mg baclofen dose (Table 2). Terminal half-life (*t*_1/2_) was consistently around 3 h, independent of dose or group (Table 2).

**Table 2:** Pharmacokinetic parameters with 95% confidence intervals for model fit.

	**Healthy controls**		**Abstinent AD**	
	**10 mg (*n* = 8)**	**60 mg (*n* = 12)**	**60 mg (*n* = 8)**	**90 mg (*n* = 6)**
*C*_max_ (ng/ml)	154.4	681.9	593.7	786.4
95% C.I.	(134.4–174.1)	(390.9–998.5)	(547.0–641.7)	(0–5,763)
*T*_max_ (min)	62	88	102	135
95% C.I.	(57–67)	(82–111)	(101–104)	(0–360)
*t*_1/2_ (h)	2.7	3.0	2.8	2.7
95% C.I.	(2.4–3.1)	(2.3–5.4)	(2.7–3.0)	(0–5,000)
*AUC* (min*ng/ml)	35,734	174,944	155,824	215,680
95% C.I.	30,604–41,555	101,060–282,511	142,744–169,777	0–1,875,126

### Pharmacodynamics-Objective Measures

#### Growth Hormone

**Figure 2:**
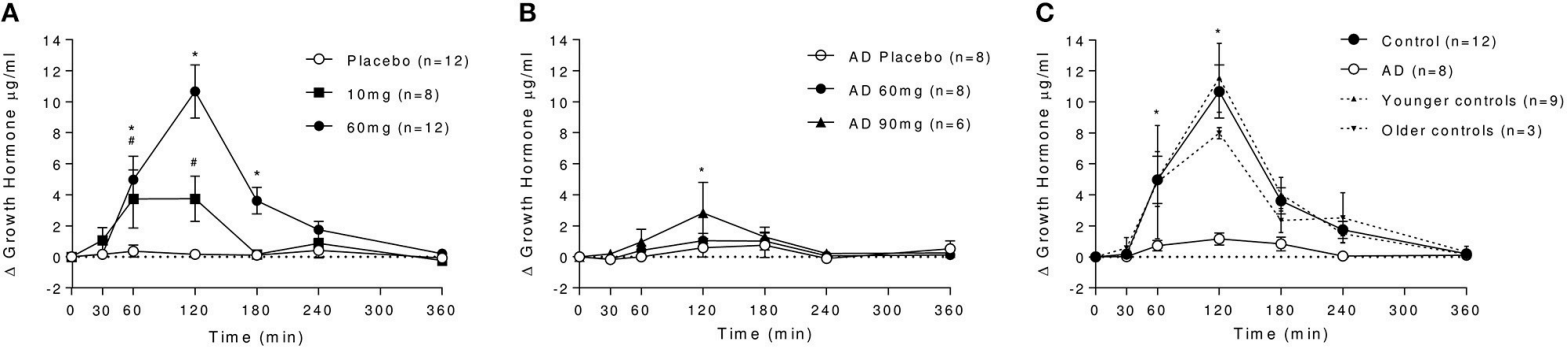
Effect of baclofen on plasma GH levels in controls and AD. Data are mean ± S.E.M. **(A)** Effect of placebo, 10 and 60 mg baclofen on GH levels in controls. A significant effect of time, dose and a dose by time interaction was observed [*n* = 8, Time: *F*_(6, 48)_ = 10.44, *P* < 0.0001, Dose: *F*_(2, 16)_ = 11.34, *P* = 0.0009, Dose × Time: *F*_(12, 96)_ = 10.16, *P* < 0.0001]. *60 mg > placebo, ^#^10 mg > placebo (*P* < 0.05, Tukey's multiple comparison test). **(B)** Effect of placebo, 60 and 90 mg baclofen on GH levels in AD. *90 mg > placebo (*P* < 0.05, Tukey's multiple comparison test). **(C)** Differential effect of baclofen on plasma GH levels in controls and AD. A significant effect of group [*F*_(1, 18)_ = 15.36, *P* = 0.001], time [*F*_(6, 108)_ = 16.35, *P* > 0.0001] and a dose × time interaction [*F*_(6, 108)_ = 10.31, *P* < 0.0001] were observed. *Control > AD (*P* < 0.05, Sidak's multiple comparison test). Differential effects on GH in younger controls (cohort 1, *n* = 9, dashed line) and older controls (cohort 2, *n* = 3, dotted line) are also depicted in **(C)**. There was no significant difference between GH levels following baclofen in younger and older control cohorts at any time point, and the significant differences between AD and both young and old controls remained (mixed ANOVA with Sidak's multiple comparison test). The average age of the younger cohort was 24.7 ± 4.7 years, compared with the older cohort (average age 52.0 ± 6.1) and the AD group (53.1 ± 8.9 years).

In controls, baclofen dose-dependently increased GH levels at 10 and 60 mg doses (Figure 2A), with peak effects measured at 120 min. Peak plasma GH levels were over 2-fold higher following 60 mg (observed *C*_max_: 10.8 ± 6.0 μg/ml) compared with 10 mg baclofen (observed *C*_max_: 4.1 ± 4.9 μg/ml). Both returned to baseline levels within 240 min. GH was unchanged after placebo. In AD, there was no significant effect of baclofen dose on GH (Figure 2B). An effect of time was observed [*F*_(6, 30)_ = 3.223, *P* = 0.015], driven by a small but significant increase in GH after 90 mg baclofen at the 120 min time-point only, where plasma levels reached 3.0 ± 4.8 μg/ml.

This marked differential effect of baclofen on GH release between groups at the 60 mg dose is depicted in Figure 2C), in which it can be observed that baclofen-induced increases in GH are absent in the AD group relative to controls. There was no significant difference between younger (*n* = 9) and older (*n* = 3) cohorts of healthy controls in their GH response to 60 mg baclofen, and there was no association between age and GH response in our control sample (*R*^2^ = 0.0671) so the absence of a response in AD group is not age-related.

#### Zig-Zag Tracking Task

**Figure 3:**
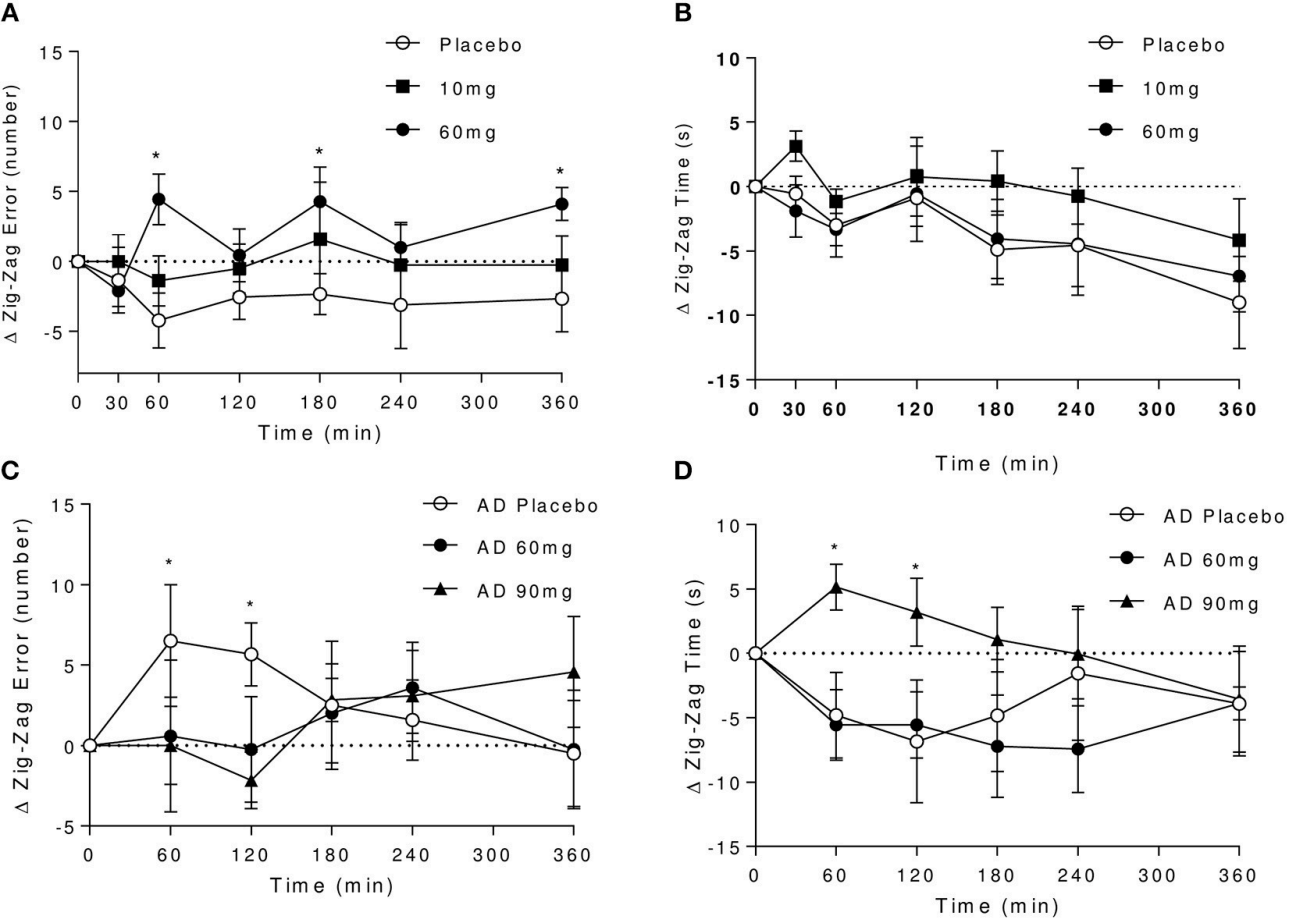
Effect of baclofen on Zig-Zag tracking task performance in controls and AD. Data are mean ± S.E.M. **(A)** number of errors following administration with placebo, 10 and 60 mg baclofen in controls. No significant time or interaction effects, trend for an effect of dose [Repeated measures ANOVA, *n* = 9; effect of dose: *F*_(2, 16)_ = 2.94, *P* = 0.08], *60 mg > placebo (*P* < 0.05, Tukey's multiple comparison test). **(B)** Time to completion after placebo, 10 and 60 mg baclofen in controls. Significant effect of time [*F*_(6, 48)_ = 4.14, *P* = 0.002], no effect of dose or interaction. **(C,D)** Number of errors and time to completion after placebo, 60 and 90 mg baclofen in AD. No overall significant dose, time or interaction effects were observed (*n* = 6), but significant effects of 90 mg dose were observed at 60 and 120 min (*P* < 0.05), **(C)** *90 mg < placebo, **(D)** ^*^placebo > 90 mg.

Optimal performance on this task is achieved through a trade-off between speed and accuracy, and is typically subject to practice effects. It can be observed that in controls under placebo, the error rate improves over the first three attempts at the task then subsequently plateaus, whilst the time to complete the task continues to shorten over the course of the whole testing period (Figures 3A,B). Under 60 mg baclofen, the relative improvement on error rate did not occur with time. Although there were no overall significant effects of dose on zig-zag task performance across the whole testing period, there was a trend for an effect of dose on error rate [*F*_(2, 16)_ = 2.94, *P* = 0.08], such that significant increases in errors were observed at 60 and 180 min post 60 mg dose relative to placebo. A significant reduction in time taken to complete the task over time was observed, independent of dose [F_(6, 48)_ = 4.14, *P* = 0.002], reflecting an overall improvement in task performance with practice in controls.

In the AD group, under placebo there were no apparent improvements in error rate with practice (Figure 3C). Indeed, an increase in the number of errors made under placebo relative to baclofen was observed at earlier time points, which coincided with reductions in time taken to complete the task (Figure 3D). There was no significant effect of time on task performance and no significant dose or interaction effects. After 90 mg, there was a significant slowing in speed of completion at 60 and 120 min post dose relative to placebo (Figure 3D), with a corresponding reduction in the number of errors made (Figure 3D). There were no significant effects of 60 mg baclofen in AD relative to placebo.

Direct comparisons between the effects of 60 mg baclofen across groups revealed no significant differences in performance (*n* = 12 and 8, data not shown), but all participants showed significantly reduced completion times with practice at this dose [effect of time; *F*_(5, 90)_ = 3.77, *P* = 0.004].

Overall this suggests differential effects of baclofen on tracking performance. The data suggest there may be an effect of 60 mg baclofen (but not 10 mg) to impair performance without sedative effects in controls, and an effect of 90 mg (but not 60 mg) to produce a slowing of performance in AD, possibly reflecting sedative component.

#### Vital Signs

In controls, there were no significant effects of baclofen on blood pressure or heart rate overall, although a significant reduction in HR in response to 10 mg baclofen was evident at high plasma baclofen concentrations (60 and 120 min). There was a significant decrease in diastolic blood pressure over time [*F*_(6, 48)_ = 8.13, *P* < 0.0001] independent of dose, and time-dependent alterations in systolic blood pressure were also observed which were in keeping with task-related activity and time of day effects [*F*_(6, 48)_ = 2.45, *P* = 0.038].

In AD (*n* = 6), there was a marginally significant dose by time interaction effect of baclofen on diastolic BP [*F*_(12, 60)_ = 1.97, *P* = 0.044]. In addition, systolic and diastolic BP were transiently increased by 60 mg baclofen at 120, 180, and 240 min relative to placebo. As was observed in controls, a significant effect of time on systolic BP [*F*_(6, 30)_ = 6.75, *P* = 0.0001] was observed.

In group comparisons between controls and AD after 60 mg baclofen (*n* = 12 and 8), a significant group by time interaction effect on HR was observed [*F*_(6, 108)_ = 3.04, *P* = 0.0087], such that HR tended to increase in controls and decrease in the alcohol group with time relative to baseline. No significant peak effects were observed in any measure.

None of the effects observed were deemed to be clinically significant.

### Pharmacodynamics-Subjective Measures

#### SHAS

**Figure 4:**
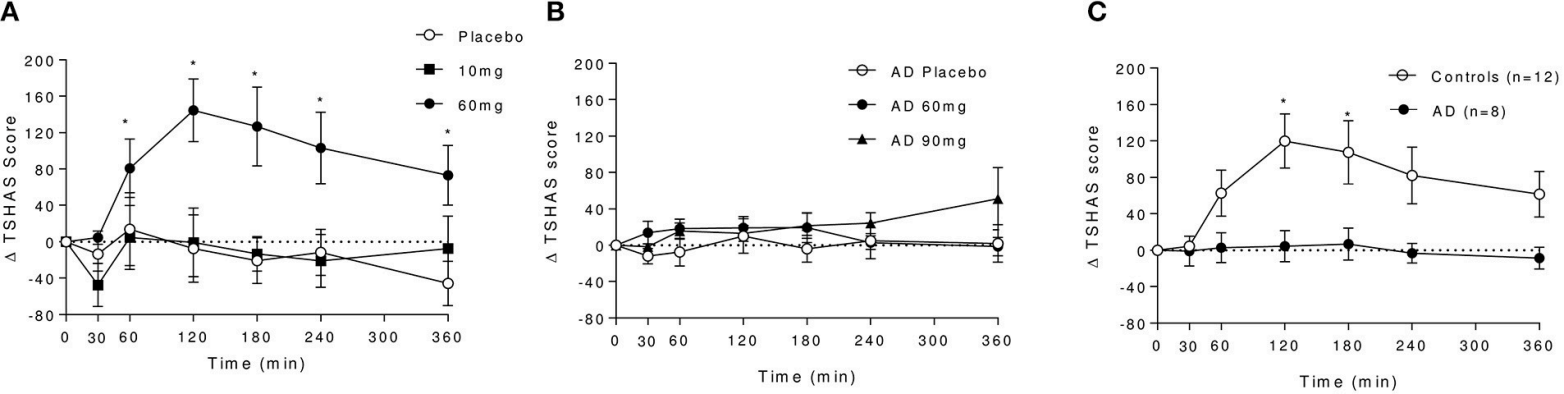
Effect of baclofen on Total SHAS (TSHAS) scores in controls and AD following placebo, 10, 60, or 90 mg baclofen. Data are mean ± S.E.M., expressed as change from baseline. **(A)** Effect of placebo, 10 and 60 mg baclofen on TSHAS in controls. Repeated measures ANOVA; Dose × Time Interaction: *F*_(12, 96)_ = 3.20, *P* = 0.0007. *60 mg > placebo (*P* < 0.05, Tukey's multiple comparison test). **(B)** Effect of placebo, 60 and 90 mg baclofen on TSHAS in AD. Repeated measures ANOVA; no significant effect of dose or time, significant dose by time interaction [*F*_(12, 60)_ = 2.37, *P* = 0.014], *90 mg > placebo (*P* < 0.05, Tukey's multiple comparison test). **(C)** Comparison of the effect of 60 mg baclofen on TSHAS in controls and AD. Mixed ANOVA; significant group effect: *F*_(1, 18)_ = 6.0, *P* = 0.025). *60 mg > placebo (*P* < 0.05, Sidak's multiple comparison test).

**Figure 5:**
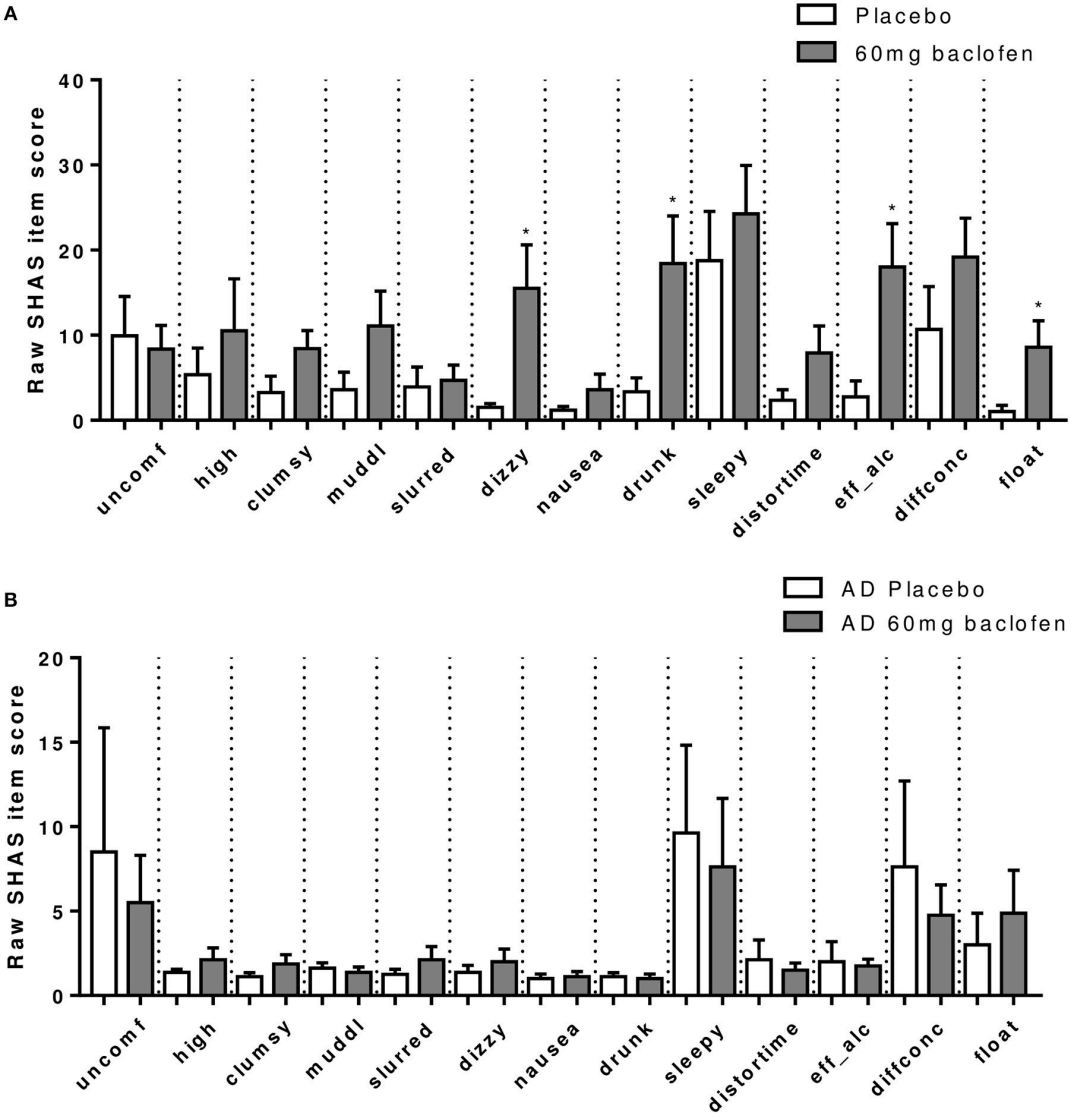
Effect of baclofen on SHAS subscores in **(A)** controls (*n* = 12) and **(B)** AD (*n* = 8) after 60 mg baclofen. Data are mean ± S.E.M. and represent the raw scores which are a breakdown of total SHAS items at 120 min post administration of 60 mg baclofen. The SHAS consists of 13 items scored 0–100. These items are “uncomfortable,” “high,” “clumsy,” “muddled,” “slurred speech,” “dizzy,” “nauseated,” “drunk,” “sleepy,” “distorted sense of time,” “effects of alcohol,” “difficulty concentrating,” and “feeling of floating”. *60 mg > placebo (*P* < 0.05).

A robust increase in total SHAS scores was observed in controls following baclofen [Dose × Time Interaction: *F*_(12, 96)_ = 3.20, *P* = 0.0007, Figure 4A], with significant effects of 60 mg compared with placebo at all time-points except 30 min. Peak effects were observed at *t* = 120 min which diminished with time but remained significant at 6 h post dose. Subscore analyses revealed that increases at *t* = 120 min in response to baclofen relative to placebo were driven primarily by items related to sensations similar to the effects of alcohol such as feeling “drunk,” “effects of alcohol,” “dizzy,” and “float” (Figure 5A), all of which were significantly increased by 60 mg baclofen. In repeated measures ANOVA analyses, these subscales also demonstrated overall significant dose and dose by time interaction effects (data not shown). Figure 5A shows the outcome of paired *t*-tests comparing the TSHAS raw scores at *t* = 120 min between placebo and 60 mg baclofen, uncorrected. A trend for a reduction in the “uncomfortable” item was observed in controls following the 10 mg dose [Dose by Time interaction: *F*_(12, 96)_ = 1.83, *P* = 0.053], which was significant at all time-points relative to placebo except 360 min.

In the AD group, no significant effect of baclofen at the 60 mg dose was observed on total TSHAS scores. A significant Dose by Time interaction was apparent [*F*_(12, 30)_ = 2.37, *P* = 0.014] which was driven by a significant effect of 90 mg relative to placebo at the 360 min time-point only. There were no significant differences in subscore items between placebo and 60 mg at peak baclofen concentrations (Figure 5B). The only subscore item which displayed significant dose effects in this group was “sleepy” [Dose by Time interaction: *F*_(12, 60)_ = 2.18, *P* = 0.024], in which there was an increase following 90 mg at the 360 min time-point (thus mirroring the overall interaction effect in total TSAS score).

Comparison of TSHAS scores (*n* = 12 and 8) revealed substantial differences between the control and AD group to 60 mg baclofen (Figure 4C). TSHAS scores were markedly lower in AD relative to controls for the duration of the study day [group effect: *F*_(1, 18)_ = 6.0, *P* = 0.025], with peak effects at 120 and 180 min. TSHAS subscore analyses revealed significant blunting of the effect of baclofen in the AD group in “drunk” and “effect of alcohol” items (significant group effects) relative to controls. Significant blunting at peak baclofen concentrations was observed in the following TSHAS items; “drunk,” “dizzy” “effect of alcohol,” “muddled,” and “difficulty concentrating” (unpaired *t*-tests, uncorrected).

#### BAES

**Figure 6:**
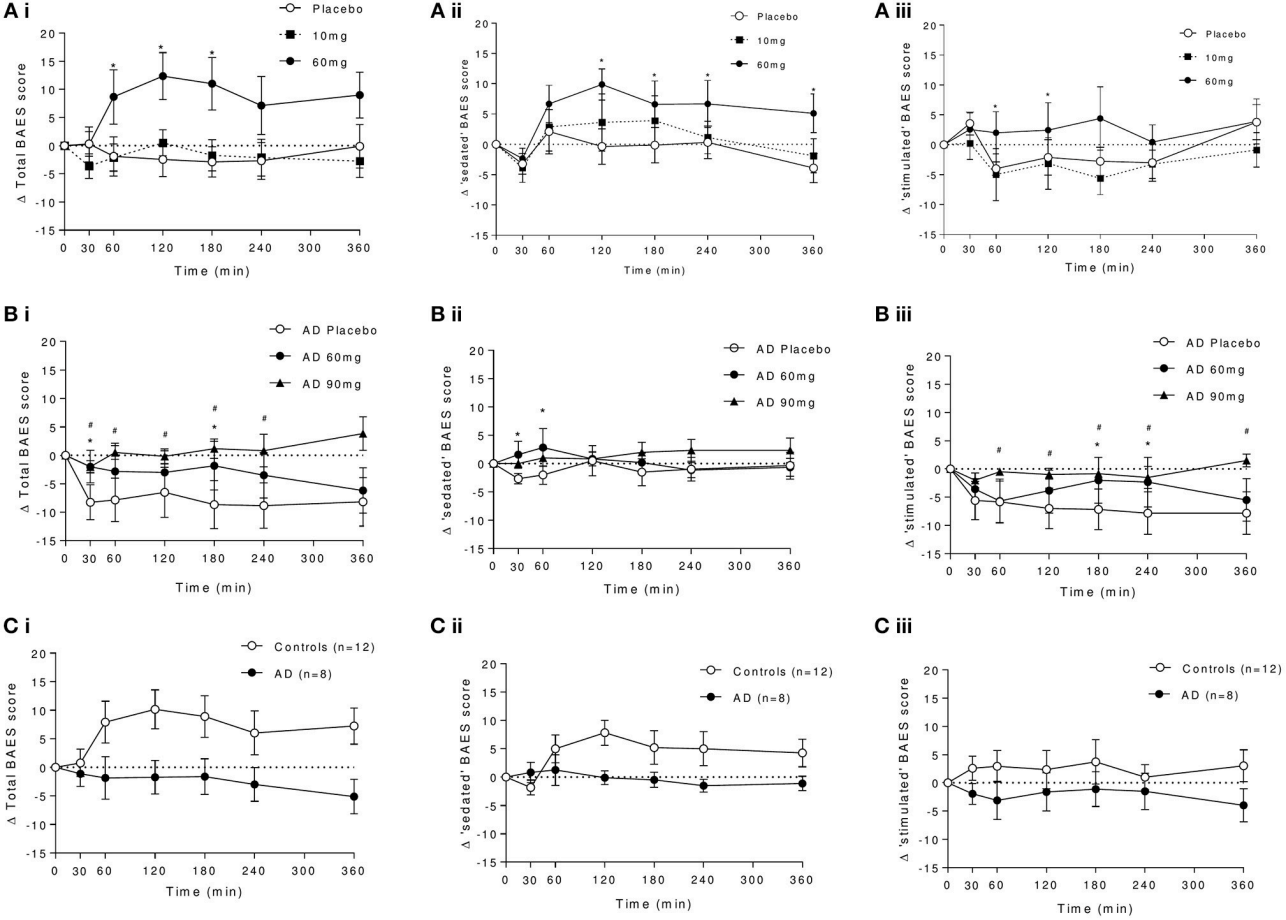
Effect of baclofen on **(i)** total, **(ii)** sedating, and **(iii)** stimulating components of the biphasic alcohol effects scale (BAES) in controls and AD. **(A)** Shows the dose response to placebo, 10 and 60 mg baclofen in controls. **(B)** Shows the dose response to placebo, 60 and 90 mg in AD. **(C)** Shows the group comparison at 60 mg baclofen (*n* = 12 controls and *n* = 8 AD) of **(i)** total, **(ii)** sedation, and **(iii)** stimulation components of the BAES. Data are mean ± S.E.M., *60 mg baclofen > placebo, ^#^90 mg baclofen > placebo, (*P* < 0.05). Scale-items contributing to the sedation effects include “difficulty in concentrating,” “down,” “heavy head,” “inactive,” “sedated,” “slow thoughts,” and “sluggish.” Scale-items contributing to the stimulation effects include “elated,” “energized,” “excited” “stimulated” “talkative,” “up” and “vigorous.” Items are scored between 0 and 8.

Significant increases in total BAES scores were observed in controls following 60 mg baclofen [Dose: *F*_(2, 16)_ = 3.85, *P* = 0.043, Interaction: *F*_(12, 96)_ = 3.06, *P* = 0.0011], with significant increases at 60, 120, and 180 min post dose relative to placebo (Figure 6Ai). This effect was driven primarily by increases in the “sedation” subscale (significantly increased at 120 and 180 min) and to a lesser extent by increases in “stimulation”'subscale (significantly increased at 60 and 180 min) after 60 mg baclofen (Figures 6Bii,iii). There were no significant difference in BAES scores after 10 mg baclofen.

In AD, there was a trend for an effect of baclofen to increase total BAES scores (Figure 6B, dose by time interaction: *F*_(12, 60)_ = 1.90, *P* = 0.053) which was driven primarily by a significant attenuation by 90 mg baclofen of time-dependent reductions in the stimulation component score following placebo at 60, 120, 180, and 240 min post dose (Figure 6Biii). There were also small but significant increases in sedation score in response to 60 mg baclofen at 30 and 60 min (Figure 6Bii).

In a direct group comparison (*n* = 12 and 8), the effect of baclofen to significantly increase total BAES scores in controls following 60 mg can be seen to be significantly blunted in AD [group effect: *F*_(1, 18)_ = 4.23, *P* = 0.05, group by time interaction: *F*_(6, 108)_ = 3.89, *P* = 0.0015, Figure 6C]. This group effect was driven by the difference in sedation, and not the stimulation component [group by time interactions: *F*_(6, 108)_ = 3.61 and 0.97, *P* = 0.0027 and 0.45, respectively].

#### VAS

In controls (*n* = 9), there was no overall effect of baclofen on VAS scores. However, a significant effect of time for both “sleepy” and “alert” factors was observed [*F*_(6, 48)_ = 4.67 and 5.66, respectively, *P* < 0.001]. Increases in subjective sleepiness as the day progressed were evident across all three doses, with corresponding reductions in alertness. In terms of peak effects, a significant reduction in alertness was observed following 10 mg baclofen at 60 min, and following 60 mg at 60, 120, and 180 min post administration, relative to placebo (*P* < 0.05, Tukey's multiple comparison test). There were no changes in VAS scores for “relaxed” or “tense.”

In AD (*n* = 6), there were no significant overall effects of time or baclofen dose on VAS measures. A dose by time interaction was observed in the “relaxed” measure [*F*_(12, 60)_ = 2.33, *P* = 0.016], primarily driven by attenuation of reductions in relaxation observed under placebo by baclofen, at peak effects: a significant increase in response to 60 mg baclofen was observed at 60, 120, 180, 240, 360 min, and in response to 90 mg at 60 and 120 min relative to placebo (*P* < 0.05, Tukey's multiple comparison test).

There were no group differences in response to 60 mg baclofen, and no other significant effects apart from an effect of baclofen to increase “sleepy” factor with time [*F*_(6, 108)_ = 2.61, *P* = 0.02], which was primarily driven by the control rather than AD group.

#### DEQ

Exploratory analyses of responses to the drinking expectancy questionnaire (DEQ) at peak baclofen effects (60, 120, and 180 min) in the older control cohort (*n* = 3) relative to AD (*n* = 8) revealed no significant group differences following 60 mg baclofen [*F*_(6, 12)_ = 10.43, *P* = 0.084]. However, the “feel some effect” factor was significantly higher than placebo at 120 min post-dose (60 mg > placebo, *P* < 0.05), Tukey's multiple comparison test, with no changes in “high,” “like,” “dislike,” or “want more” factors. Conversely in AD, there was a tendency to “dislike” the effects of 60 mg baclofen at 60 min post dose, with a non-significant increase in “feel some effect” factor at 120 min, instead reporting significant increases in “high” and “I would like more” at this time point.

#### Guess Analysis

Subjects were asked to guess which doses they had received at each visit. In controls, 8 of 9 (89%) receiving the 60 mg dose guessed correctly that they had received baclofen. Fewer correctly guessed the 10 mg dose; 5 of 8 (63%) receiving 10 mg baclofen correctly guessed they had received baclofen. 5 of 9 (56%) receiving placebo correctly guessed that they had received placebo, with only one confusing this with the high (60 mg) baclofen dose. These data suggest that the effects of the lower dose were more subtle.

In AD, 5 of 8 (63%) correctly guessed baclofen when they had received 60 mg (i.e., the same proportion to controls receiving 10 mg), whereas the vast majority (5 of 6, 83%) were able to correctly discriminate 90 mg, pointing to greater drug effects at this dose. 4 of 8 (50%) guessed correctly on the placebo day.

### Pharmacodynamics-EEG Measures

**Figure 7:**
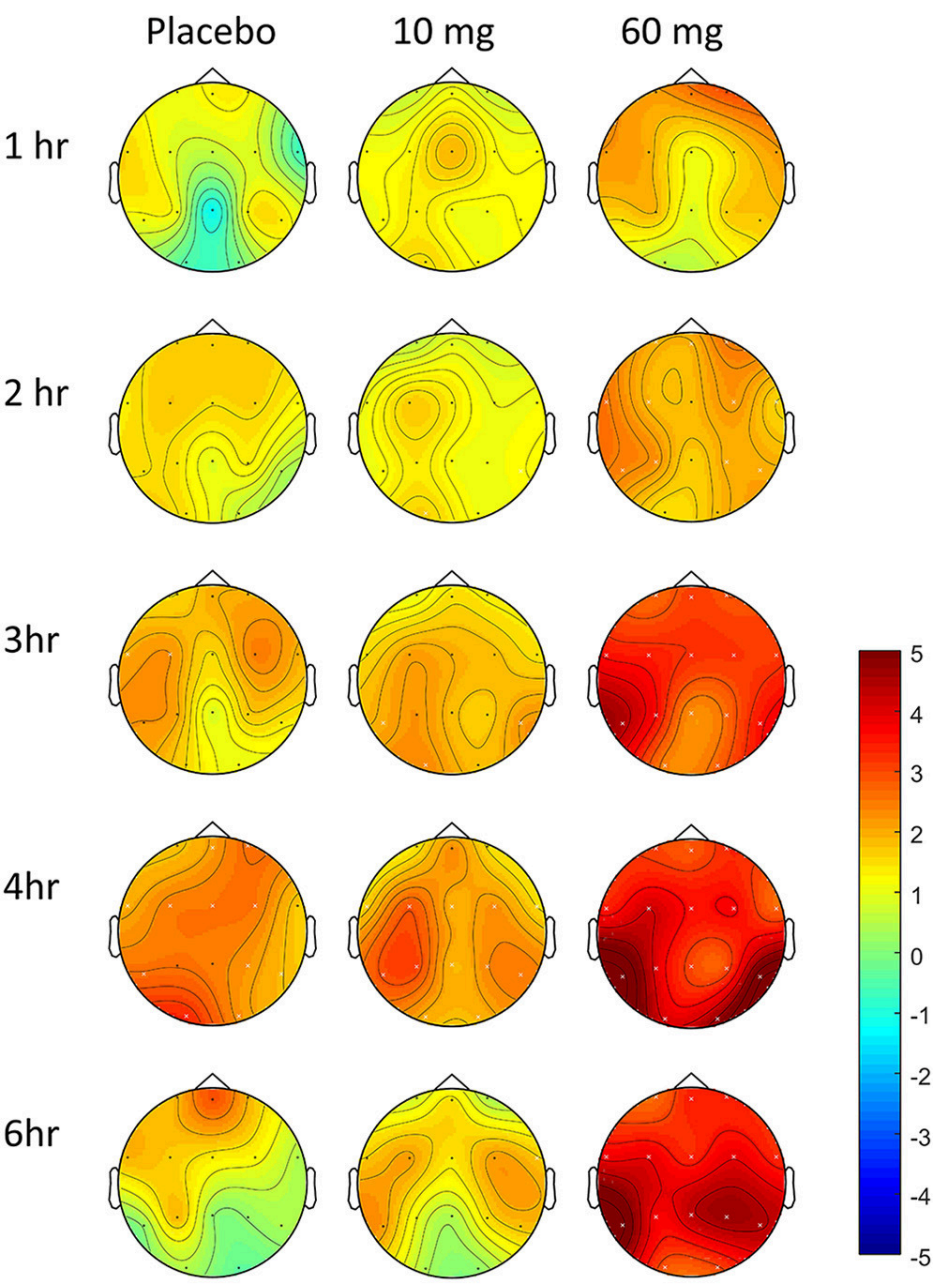
Effect of baclofen on change in theta power over time in controls, relative to baseline. Data are theta power (4–8 Hz EEG activity), change from baseline comparisons after placebo (*n* = 7), 10 mg baclofen (*n* = 5) and 60 mg baclofen (*n* = 7) at five time points following dosing (eyes shut). Pre-intervention baseline spectra were subtracted from spectra at each post-intervention time-point (1, 2, 3, 4, and 6 h post baclofen) to provide statistical maps (paired *t* statistics). Red indicates relatively more power following baclofen, and blue indicates relatively less power. Black dots represent location of electrodes. Differences relative to baseline were tested using permutation testing; white crosses represent channels with significant differences relative to baseline (FDR corrected).

**Figure 8:**
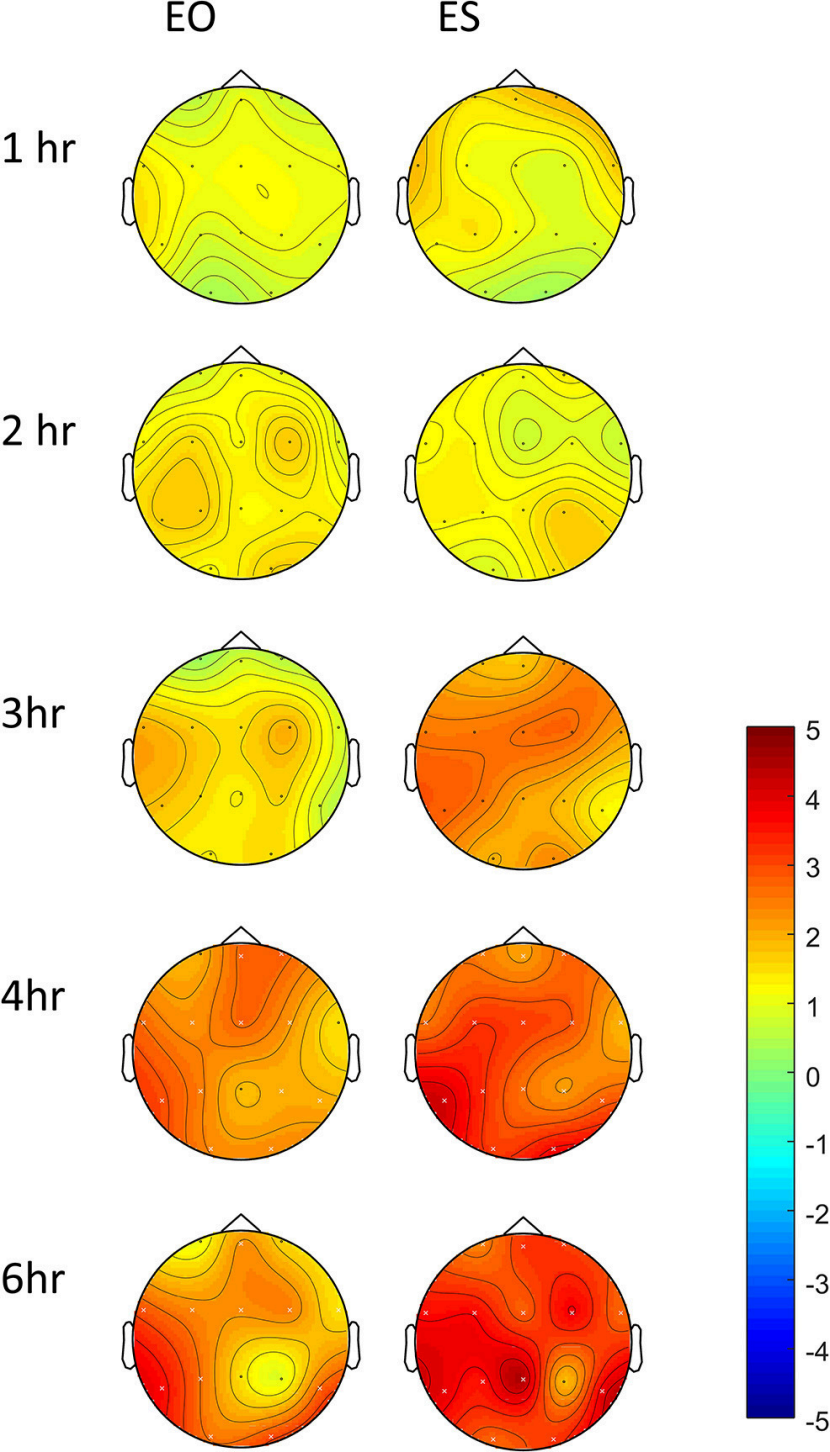
Differences in change-from-baseline theta power between placebo and 60 mg baclofen, shown with both eyes open (EO) and eyes shut (ES) at 5 time points in controls (*n* = 7). Contrasts were performed between placebo and baclofen (t statistics of difference between the difference at baseline) to create one image per time point. This represents a subtraction of column 1 (placebo) from column 3 (60 mg baclofen) as depicted in Figure 7. Red indicates relatively more power following baclofen relative to placebo. Electrodes with significant differences between placebo and baclofen are shown with a white cross, corresponding to *p* < 0.05, FDR corrected.

**Figure 9:**
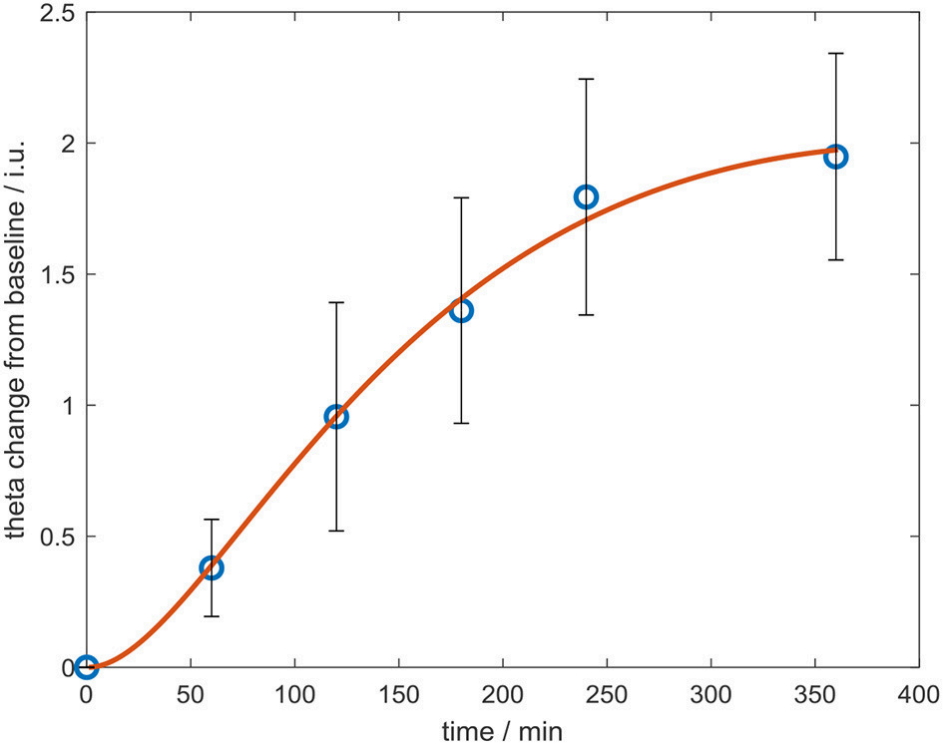
Change in whole-brain theta power from baseline over the course of the experiment, after the 60 mg dose of baclofen, with eyes shut. Data are mean ± S.E.M. The data were fitted using a compartmental model (red line).

In controls, increased theta power was observed as the study day progressed in all three conditions (Figure 7), but was more pronounced following the 60 mg baclofen dose, particularly posteriorly and this change was reliably and consistently observed across participants. In a subsequent comparison between 60 mg baclofen and placebo, significant increases in theta were evident at 4 and 6 h post administration in both eyes open and eyes closed conditions (Figure 8). There were no significant differences between 10 mg and placebo. Changes in theta power from baseline were well fitted by compartmental modeling over the time course of the experiment, using the baclofen plasma data as the input function (Figure 9). The time-course of the effect was different to that of plasma baclofen levels and subjective effects of baclofen. Baclofen concentration-effect plots demonstrate that while plasma baclofen and subjective or GH effects follow a similar time course (Figures 1, 4), theta activity appears delayed, and continues to rise long after plasma baclofen *C*_max_, with highest values at the final time point (360 min). No later recordings were made.

In AD, large variations in theta activity (and other bands) were observed between participants, both at baseline and on placebo days, possibly due to age. We were unable to reliably model the data, and therefore group level analyses did not reveal consistent findings across any frequency bands (data not shown).

## Discussion

We have shown here in this unique pharmacokinetic-pharmacodynamic study, that baclofen induced objective and subjective effects are substantially blunted in the AD group compared with healthy controls, in the absence of any significant differences in pharmacokinetics. Our data therefore suggest that lower GABA-B receptor sensitivity is present in alcohol dependence.

The pharmacokinetic profile of baclofen in our healthy control and alcohol dependent cohorts is broadly consistent with that reported previously, with peak plasma levels evident at about 1hr after dosing, and a half-life of approximately 3 h (30–32, 58). There is some variation in pharmacokinetic indices between studies which may be due to dose, age and gender of participants, different tablets, absorption, blood sampling times and methods of analysis of baclofen plasma levels. The overall lack of clinically significant changes in blood pressure and heart rate suggests that baclofen is safe, from a cardiovascular perspective, at these doses.

We found that the pharmacokinetic profile of 60 mg of baclofen was similar in healthy controls and abstinent alcohol dependence. Indeed, there were no significant differences in any PK parameter between groups. Two previous studies have similarly reported that the PK does not differ markedly between alcohol dependence and healthy controls (34, 59). These studies reported the baclofen PK effects following treatment with 15–250 mg daily, in divided doses. In both, a linear relationship was found, even at higher (>120 mg) doses, but substantial inter-individual variability was evident, which could not be attributed to age, gender, weight, smoking status, renal or liver function. It was suggested the variability might be responsible for differences in clinical response. Consistent with these studies, we observed greater variability in plasma baclofen levels in our alcohol dependent group at 90 mg compared with 60 mg. One likely and important difference between these clinical studies and our lab-based study is that our participants had been abstinent from alcohol for a considerable number of months, from 8 to 72, whilst the drinking status was not clear in the clinical samples.

Our AD participants were older than the healthy controls. However, we found no relationship between age and peak plasma levels of baclofen, and no difference in PK parameters between the younger and older control cohorts. Additionally, others have reported no difference in *T*_max_ or total plasma baclofen levels in older (69–81 years) adults compared with younger controls (23–53 years), although elimination times of baclofen were slower in the older participants which was likely due to impaired renal function (58). Our alcohol dependent participants were all healthy with normal renal and hepatic function.

We were only able to compare the effects of 60 mg baclofen between the two groups since the limited effects of the 10 mg dose in healthy controls meant that we did not investigate this dose in the AD group, and the pronounced sedation induced by 90 mg in our first healthy control precluded further analysis or comparisons at this dose.

The subjective effects of 60 mg of baclofen in our healthy controls were as expected, with marked increases seen in both the SHAS and BAES scales, and these effects were dose-dependent. 10 mg of baclofen resulted in more limited subjective effects, whereas 90 mg resulted in profound sedation such that the participant could not complete the protocol. The time-course of these observations are consistent with other studies using similar doses of oral baclofen (36). Following 60 mg baclofen, controls reported a number of subjective effects which are primarily sedative in nature, including a selective increase in sedation scores on the BAES and also drunkenness, dizziness, floating and other effects similar to those of alcohol in the SHAS.

Conversely, in alcohol dependent participants the 60 mg dose did not result in significant changes in any subjective measures. At 90 mg, there was a suggestion of mild increases in sleepiness as measured by the SHAS, and attenuation of the stimulation component of the BAES but no direct evidence of significant increases in subjective sedation. These data are consistent with the vast majority of control, but not AD, participants being able to correctly identify when they had received 60 mg baclofen relative to placebo in the guess analysis. The AD group could only reliably guess they had received baclofen at the higher 90 mg dose. Interestingly, in clinical trials of baclofen in alcohol dependence, dizziness is one of the most common side-effects experienced from a range of doses but was not reported here, after a single 60 or 90 mg dose of baclofen.

Sedation is a commonly cited side-effect and limits tolerability in some patients, whereas others welcome it and find some sedation acceptable (17, 29). In controls, subjective sedation was demonstrably higher following 60 mg baclofen, with the increases observed in the BAES primarily related to increases in the sedation rather than stimulation subscale. It is therefore notable that limited sedation was seen in our abstinent alcohol dependent individuals, even after 90 mg. In previous studies, baclofen has been reported to increase sedation in a lab-based study in heavy drinkers [40 mg, 80 g; (36)], but baclofen (30 mg/d) alone did not increase sedation in a study of alcohol dependent individuals (60). It therefore appears that alcohol dependent individuals are less sensitive to the sedative effects of baclofen alone. This is consistent with clinical experience where those who do not experience sedation are able to tolerate “higher” doses of baclofen.

Performance on the zig-zag task, which we chose as an objective measure of sedative effects of drugs, was interestingly not significantly different overall between healthy controls and abstinent alcohol dependent participants after 60 mg of baclofen. However, there was some evidence of reduced accuracy following 60 mg in controls, but not in AD. There was also evidence of slowing in AD following 90 mg baclofen, pointing to a possible psychomotor retardation effect at this dose. The slowing in speed of completion was accompanied by a reduction in error rate suggesting that participants were engaging in a compensatory trade-off, such that the reduced speed permitted fewer errors. This could be important, as although participants were not overtly sedated, and higher doses were better tolerated, there may still be risks associated with altered motor responses in alcohol dependence e.g., driving. The pattern of performance in healthy controls was also different since their performance consistently improved with practice. They appeared to adjust their speed to achieve higher levels of accuracy if their previous performance was poor, an adaption which was not clearly observed in abstinent alcohol dependent participants.

The finding of increased EEG theta activity in controls is supported by previous clinical studies of sleep and EEG effects after baclofen administration (35, 37). Badr et al showed increased theta activity during wakefulness following 2 days of baclofen (30 mg, bid), whereas Vienne et al showed increased theta activity during a daytime nap following acute baclofen. Increased theta was also apparent during the subsequent night time sleep period, alongside increases in first-episode slow-wave sleep, total sleep time and decreases in sleep onset latency, at baclofen doses equivalent to approximately 30 mg (37). A study combining EEG and transcranial magnetic stimulation (TMS) also indicates effects of baclofen at the level of the cortex (61). Previous preclinical studies have similarly shown hypersomnia following baclofen administration in rats (62). Interestingly, this effect was not attributed to a GABA-B specific mechanism, since GABA-B1 and GABA-B2 knock out animals also displayed similar effects.

Given the concerns raised about potential abuse liability of baclofen it is interesting that, unlike the controls, our abstinent alcohol dependent participants did not report significant “alcohol-like” or “drunk” effects on the SHAS following 60 or 90 mg of baclofen. However on the drug expectancy questionnaire, although there were no overall significant effects, there was a suggestion of increased “high” and “liking” effects after the 90 mg dose. Anecdotally, several participants stated that they enjoyed the effects, and likened them to those of opiates or benzodiazepines. This has potential implications for abuse liability, particularly at higher doses, and requires further study. A study in heavy social drinkers similarly showed that unlike alcohol, baclofen (0, 40, 80 mg) did not significantly increase “high,” “drug-liking,” or “stimulation”, though at the highest dose “good drug effect” and “elevated mood” were reported (36).

Together with our data, this suggests that baclofen is not experienced as “alcohol-like,” and therefore may not be substituting for alcohol, as previously suggested as a potential mechanism underlying its efficacy in alcohol dependence (63). It should be noted however that some of the evidence supporting a substitution model comes from studies where alcohol and baclofen are combined, rather than the effects of baclofen alone. In that situation baclofen appeared to enhance “intoxication” ratings and “feeling high” from alcohol, though no reduction in alcohol consumption was observed (60).

Concerning objective effects, baclofen resulted in dose-dependent increases in GH levels which were notably absent in our abstinent alcohol dependent participants. Others have previously shown similar blunting (40–44). However, our study shows that such blunting is still present even after a high (90 mg) dose of baclofen. It also coincides with an absence of subjective effects, and PK is demonstrably no different between groups. Further it is clear from our data, and from others, that such blunting persists in to longer-term abstinence (42). It is of considerable interest that blunted baclofen-induced GH release has also been reported in opiate dependent individuals who were abstinent for a few days, and also 2 months later (64). Together this suggests that lower sensitivity to baclofen, and by extension GABA-B receptor sensitivity, might be a trait marker of addiction and play a fundamental role in the addiction process. It is not clear whether chronic alcohol or drug exposure results in this lower sensitivity. Therefore it would be interesting to assess whether baclofen induced GH levels is altered in those at high risk of addiction, such as individuals with a positive family history. It is also worth noting that GH responses to noradrenaline challenge are also blunted in alcohol dependence (65), both during withdrawal and in abstinence. These findings suggest that deficits in neurotransmitter receptor mediated endocrine responses, including those mediated by GABA-B receptors, are a feature of alcohol dependence and may persist for some time.

Our results suggest there may be a delayed component to the baclofen response; we observed short delays in the subjective response to baclofen relative to the PK profile (TSHAS and BAES effects peaked at approximately 2–3 h after dosing whilst *T*_max_ occurred at approx. 90 min), but the time-course of the EEG effects was much longer; theta activity remained elevated at the final 6 h time point, with no sign of dissipation. Preclinical studies indicate delayed central effects of baclofen including toxicity response (66), anti-nociception (67) and EEG (62, 68). There are a number of possible explanations for delayed effects including slow drug accumulation in CNS, slow equilibrium kinetics, and/or involvement of non-GABA-B dependent or downstream receptor mechanisms.

Our data showing blunted objective and subjective responses to baclofen in abstinent alcohol dependent individuals strongly suggests altered GABA-B sensitivity, but the underlying mechanism(s) cannot easily be characterized in the absence of tools such a PET ligand or GABA-B antagonist for use in man. Our findings could result from a range of different mechanism(s) such as a reduction in GABA-B receptor function. This could be brought about by a reduction in receptor number, reduced G-protein coupling or altered internalization or trafficking mechanisms (69–71). Preclinical studies have shown that chronic ethanol exposure produces neuroadaptations in GABA-B receptor function, for example by reducing the sensitivity of GABA IPSPs in the central amygdala (72), or reducing presynaptic modulation of GABA release in hippocampus (73). Alternatively, dysregulation of one of the “downstream” neurotransmitter systems that GABA-B receptors modulate could be involved. For instance, it is well known that alcohol impacts on a wide range of neurotransmitter receptor systems that GABA-B modulates including GABA-A, dopamine, noradrenaline and glutamate (9, 74). It is possible that genetic factors may contribute to the variability in PK and PD effects of baclofen. For example a polymorphism in the ABC transporter gene A*BCC9* is associated with greater clearance of baclofen (75), and the *GABBRI* polymorphism, rs29220, has been reported to modulate response to baclofen, with CC carriers deriving greater benefit with fewer side-effects compared with G-carriers (76, 77). Unfortunately we did not genotype our participants.

There are several study limitations that are worth noting. The groups were not well matched for age. This was partially mitigated by the addition of the second older control cohort (*n* = 3) which were found not to differ from the younger control cohort on any PK or PD measure. In addition, there was no association between age and any PK or PD measure in the control sample. There is also no evidence of a relationship between age and baclofen response in the clinical AUD or spasticity literature. Although circulating basal GH levels are known to decline with age, we found that increased GH release following baclofen challenge did not change significantly with age in our control sample. We studied longer-term abstinent alcohol dependent individuals who were able to complete our protocol safely in the community. We acknowledge that this population does not necessarily reflect those currently in treatment services. In clinical practice, baclofen is started soon after alcohol detoxification, or even whilst still drinking. Nevertheless our participants were still at risk of relapse, and indeed some did subsequently relapse (though not related to the study). The fact that lower baclofen sensitivity was seen even after a considerable period of abstinence requires further investigation as to its implications. Our sample size is small, but commensurate with other PK-PD and similar challenge studies where effect sizes are often large (as observed here). Whilst we observed no relationship between age or months abstinent and any PK or PD measure, the sample was too small to adequately explore such relationships, or to assess, for example, whether alcohol exposure was related to baclofen sensitivity. Despite the small sample, the effect size of the group differences observed in subjective and GH response to baclofen were substantial. For example Cohen's d was 2.19 for peak GH levels following 60 mg baclofen at 120 min post-dose. This makes it unlikely that smaller contributions made by age, or other factors, would change the study outcome.

In summary we have shown blunted sensitivity to baclofen in abstinent alcohol dependent individuals compared with healthy controls. This adds to the growing evidence about dysregulated GABA-B system in addiction, though the underlying mechanism is not well characterized. This lower sensitivity likely contributes to the fact that “high” doses of baclofen are tolerated in alcohol dependence, and are proposed as necessary for the effective treatment of alcohol dependence. This study also has important implications with regard to the choice of baclofen dose in future studies in addiction. It may also serve those investigating the potential of other GABA-B agonists or positive allosteric modulators in meeting the immense unmet need in treating addiction.

## Author Contributions

CD and LP contributed equally to this manuscript. CD, SW, DN, and AL-H contributed to conception and design of the study. LP, CD, ST, AV, IM, and TJ were involved in data collection and collation. SP, LN, and RC performed the baclofen plasma analysis. JM performed data modeling. CD, SW, JM, and SM performed the EEG analyses. CD and LP performed all other data analyses. LP wrote the first draft of the manuscript, AL-H wrote sections of the manuscript. All authors contributed to manuscript revision, read and approved the submitted version.

### Conflict of Interest Statement

AL-H has received Honoraria paid into her Institutional funds for speaking and Chairing engagements from Lundbeck, Lundbeck Institute UK, Janssen-Cilag; received research grants or support from Lundbeck, GSK; consulted by Silence and also consulted by but received no monies from Britannia Pharmaceuticals, GLG, Opiant and Lightlake. DN sits on advisory Boards for Lundbeck, MSD, Nalpharm, Orexigen, Shire, MSD, Mundipharma, Ranvier, Indivior, D&A pharma, has received speaking honoraria (in addition to above) from BMS/Otsuka, GSK, Lilly, Janssen, Servier, AZ, Martindale, is a member of the Lundbeck International Neuroscience Foundation, Chair Campus editorial board, has received grants or clinical trial payments from P1vital, MRC, NHS, Lundbeck, has share options in P1vital, Alcarelle, and is Director Equasy Enterprises. The remaining authors declare that the research was conducted in the absence of any commercial or financial relationships that could be construed as a potential conflict of interest.
